# Alpha-arylphorin is a mitogen in the *Heliothis virescens* midgut cell secretome upon Cry1Ac intoxication

**DOI:** 10.7717/peerj.3886

**Published:** 2017-10-03

**Authors:** Anais Castagnola, Jerreme Jackson, Omaththage P. Perera, Cris Oppert, Shigetoshi Eda, Juan Luis Jurat-Fuentes

**Affiliations:** 1 Department of Entomology and Plant Pathology, University of Tennessee, Knoxville, TN, United States of America; 2 Genome Science and Technology Program, University of Tennessee, Knoxville, TN, United States of America; 3 Southern Insect Management Research Unit, United States Department of Agriculture-Agricultural Research Service, Stoneville, MS, USA; 4 Department of Forestry, Wildlife and Fisheries, University of Tennessee, Knoxville, TN, United States of America; 5 Current affiliation: ManTech International Corporation, Herndon, VA, United States of America; 6 Current affiliation: Oklahoma State University, Department of Microbiology and Molecular Genetics, Stillwater, OK, United States of America; 7 Current affiliation: Bayer CropScience, Morrisville, NC, United States of America

**Keywords:** Arylphorin, *Bacillus thuringiensis*, *Heliothis virescens*, Regeneration, Stem cell, Midgut, Cry1Ac

## Abstract

Insecticidal crystal (Cry) proteins produced by the bacterium *Bacillus thuringiensis* (Bt) target cells in the midgut epithelium of susceptible larvae. While the mode of action of Cry toxins has been extensively investigated, the midgut response to Cry intoxication and its regulation are not well characterized. In this work, we describe the secreted proteome (secretome) of primary mature midgut cell cultures from *Heliothis virescens* larvae after exposure to Cry1Ac toxin compared to control buffer treatment. The Cry1Ac-induced secretome caused higher proliferation and differentiation and an overall reduction in total cell mortality over time in primary *H. virescens* midgut stem cell cultures when compared to treatment with control buffer secretome. Differential proteomics identified four proteins with significant differences in abundance comparing Cry1Ac-treated and control secretomes. The most significant difference detected in the Cry1Ac secretome was an arylphorin subunit alpha protein not detected in the control secretome. Feeding of purified alpha-arylphorin to *H. virescens* larvae resulted in midgut hyperplasia and significantly reduced susceptibility to Cry1Ac toxin compared to controls. These data identify alpha-arylphorin as a protein with a new putative role in the midgut regeneration process in response to Cry1Ac intoxication and possibly pathogen/abiotic stress, identifying alpha-arylphorin as a potential gene to target with insecticidal gene silencing for pest control.

## Introduction

Insecticidal Cry proteins synthesized by the bacterium *Bacillus thuringiensis* (*Bt*) are used in pesticides and produced by transgenic crops to control destructive lepidopteran and coleopteran crop pests (Sanchis, 2011). The specificity of these Cry toxins is mostly determined by their binding to specific receptors on the brush border membrane of the insect intestinal epithelium (reviewed in Adang, Crickmore & Jurat-Fuentes, 2014). Binding to these receptors is conducive, in most cases, to enterocyte death and subsequent disruption of the gut epithelial barrier, allowing *Bt* and resident bacteria to invade the hemocoel and cause septicemia (Broderick et al., 2009; Raymond et al., 2009). However, it has long been established that lepidopteran larvae can recover from exposure to Cry toxins (Dulmage & Martinez, 1973; Nishiitsutsujiuwo & Endo, 1981; Sutherland, Harris & Markwick, 2003), and that recovery depends on a midgut regenerative response (Chiang, Yen & Peng, 1986; Spies & Spence, 1985). Moreover, an enhanced midgut regenerative response has been proposed as a resistance mechanism to Cry1Ac toxin in selected strains of *Heliothis virescens* (Forcada et al., 1999; Martínez-Ramírez, Gould & Ferré, 1999), highlighting the importance of this defensive mechanism in determining susceptibility to Cry toxins. However, information on the molecular regulation of this midgut healing response to Cry toxins in insects is very limited.

The most detailed information on the response to Cry intoxication has been obtained in the nematode *Caenorhabditis elegans*. In this organism both the MAPK p38 and c-Jun N-terminal kinase pathways have been reported as important to the defense response against Cry5B toxin (Huffman et al., 2004). A role for the p38 kinase in midgut defense against Cry1Ab has also been proposed in the lepidopteran *Manduca sexta* (Cancino-Rodezno et al., 2010). In *Bombyx mori* larvae, the JNK and JAK-STAT pathways were found to be up-regulated in the early response to Cry1Aa intoxication (Tanaka, Yoshizawa & Sato, 2012). Subtractive hybridization libraries and custom microarrays detected a down-regulation of metabolic enzymes and up-regulation of genes involved in detoxification, stress, or immune responses after intoxication of *Choristoneura fumiferana* and *M. sexta* larvae with Cry1Ab protoxin (Meunier et al., 2006; Van Munster et al., 2007). Proteomic analyses of Cry intoxication in the coleopteran model *Tribolium castaneum* also detected down-regulation of metabolic and up-regulation of defensive genes (Contreras, Rausell & Real, 2013a) and identified the hexamerin apolipophorin III as involved in the immune response to Cry3Ba intoxication (Contreras, Rausell & Real, 2013b). Similar trends have been reported in larvae of *Spodoptera exigua* (Herrero et al., 2007) and *Spodoptera frugiperda* (Rodriguez-Cabrera et al., 2008) challenged with Cry1Ca toxin. In the case of *S. exigua*, specific members of a family of proteins responding to pathogens (REPAT) and arylphorin genes were found to be up-regulated in response to intoxication with a *Bt*-based pesticide (Hernández-Martínez et al., 2010). This activation was constitutive in larvae from a strain of *S. exigua* resistant to the *Bt*-pesticide. Up-regulation of arylphorin precursor was also found in a Cry1Ab-resistant compared to a susceptible strain of *Diatraea saccharalis* (Guo et al., 2012). In contrast, exposure of *S. exigua* to a *Bt* toxin (Vip3Aa) with a distinctly different mode of action compared to Cry toxins, or exposure of *Lymantria dispar* larvae to a commercial *Bt* pesticide resulted in reduced arylphorin subunit alpha expression (Bel et al., 2013; Sparks et al., 2013). Although alpha-arylphorin has been previously shown to induce midgut stem cell proliferation (Hakim et al., 2007), the specific functional roles of REPAT and arylphorin proteins in midgut regeneration after Cry intoxication have yet to be elucidated.

Primary midgut cell cultures from lepidopteran larvae have been used as an *in vitro* model to study the molecular cues directing midgut regeneration (Hakim, Baldwin & Smagghe, 2010), and are capable of regeneration after intoxication with *Bt* toxins (Loeb et al., 2001b). A number of peptidic midgut proliferation and/or differentiation factors (MDFs) from mature cell conditioned media and hemolymph have been reported (reviewed in Hakim, Baldwin & Smagghe, 2010). One of these MDFs (MDF1) was localized to mature midgut cells upon Cry intoxication (Goto et al., 2001), yet its role in midgut healing has not been experimentally demonstrated.

Given that healing regulatory factors are secreted by stressed midgut cells, we hypothesized that proteomic analysis of the subproteome of secreted proteins (secretome) would allow the identification of proteins involved in the midgut response to injury. While midgut subproteomes from the midgut lumen (Pauchet et al., 2008) and peritrophic matrix (Campbell et al., 2008) have been characterized in *Helicoverpa armigera* larvae, the lepidopteran midgut cell secretome and its alteration during Cry1Ac intoxication has not been previously studied. We report the characterization and comparison of secretomes from *H. virescens* primary mature midgut cell cultures after treatment with activated Cry1Ac toxin versus control treatments to identify potential candidate proteins and test their involvement in regulating the gut regenerative response.

## Materials and Methods

### Bacterial toxin

*Bacillus thuringiensis* var. *kurstaki* strain HD73 producing Cry1Ac toxin was obtained from the *Bacillus* Genetic Stock Center (BGSC, Columbus, OH, USA). Bacterial culturing, toxin activation and purification were as described elsewhere (Perera et al., 2009). Purity of activated toxins was assessed by SDS-10% PAGE (Fig. S1), and protein concentration quantified using the Coomassie Plus Protein Assay (Pierce, Waltham, MA, USA) with bovine serum albumin (BSA) as standard. Purified toxin samples were kept at −80 °C until used (less than six months).

### Insects and toxin feeding

Eggs from the Cry1Ac-susceptible YDK strain of *H. virescens* (Gould, 1995) were kindly supplied by Dr. Fred Gould (North Carolina State University). Upon hatching, larvae were reared on artificial diet (Bio-Serv, Flemington, NJ, USA) at 28 °C on an 18L:6D photoperiod.

### Establishment of primary midgut cell cultures

All dissections and transfers were performed in the sterile environment of a biosafety cabinet. Preparation and establishment of primary midgut cell cultures were done following protocols described previously (Castagnola, Eda & Jurat-Fuentes, 2011). Briefly, midguts of fourth instar *H. virescens* larvae were dissected and cleaned of food, peritrophic matrix, and Malphigian tubules using forceps and rinsed in sterile Ringer’s (Barbosa, 1974) containing 0.5% (v/v) gentamicin (Invitrogen, Carlsbad, CA, USA), 0.1% bleach, and 1× antibiotic/antimycotic (Gibco 15240-062; Gibco, Grand Island, NY, USA). Incubation media was prepared by mixing working Grace’s (supplemented Grace’s Insect Medium [Invitrogen] containing 1× antibiotic/antimycotic and 0.1% gentamicin) in sterile Ringer’s solution in a 3:1 ratio (Loeb, Brandt & Birnbaum, 1984). For each primary cell culture preparation, five pools of five to six cleaned midguts were cut in sections and incubated in 2 ml of incubation media for 90 min at room temperature. After incubation, the midgut tissue was homogenized by carefully pipetting up and down and then sieved through 70 µm cell strainers (BD Biosciences, NJ) into a sterile 50 ml conical tube. Tubes were centrifuged (400× g for 5 min at 4 °C) and the supernatants discarded. The pellet in each tube containing mature midgut cells and midgut stem cells was resuspended in 1 ml of working Grace’s media.

Stem cells were separated from mature cells using a density gradient (Loeb & Hakim, 1999). Briefly, samples were overlaid on 3 ml of Ficoll-Paque (GE LifeSciences, Piscataway, NJ, USA) in a 15 ml conical tube, and then centrifuged (600× g for 15 min at 4 °C). After centrifugation, stem cells were collected from the top 0.99 ml, the intermediate 2.75 ml containing tissue debris were discarded, and the mature cells were collected in the pellet. Ficoll-Paque was eliminated from stem and mature cell samples by washing twice with incubation media (600× g for 5 min at 4 °C). Final stem and mature cell pellets were suspended in 0.35 or 1 ml, respectively, of working Grace’s. Stem and mature cell samples that were prepared simultaneously were pooled and the number of cells counted using a hemocytometer (Bright-Line, Horsham, PA). Using this procedure, we reproducibly obtained approximately 8 × 10^5^ stem cells and 1 × 10^7^ mature cells from 30 *H. virescens* larvae. Cells were counted in a hemocytometer and diluted to 4 × 10^5^ cells/mL (stem cells) or 3.5 × 10^6^ cells/mL (mature cells) with working Grace’s, and kept in a sterile incubator at 26 °C.

### Preparation of midgut cell secretomes

Purified mature midgut cells in working Grace’s were seeded (3.5 × 10^6^ cells) in individual wells of a 12-well culture-treated plate (Corning, Corning, PA, USA). The Cry1Ac toxin concentration (1 µg/ml) used as treatment to induce the midgut secretome was chosen based on its ability to induce low cytotoxicity (percent mortality 15.84 ± 0.29 compared to 8.09 ± 0.99 in buffer treatment) as measured by trypan blue staining of primary *H. virescens* mature cell cultures treated for 18 h at 26 °C. The purified Cry1Ac toxin (1 µg/ml) or the corresponding volume of control buffer (20 mM TRIS/HCl, 0.3 M NaCl pH 8.0), were added to the cultures and incubated for 18 h at 26 °C. After incubation, media supernatant containing the proteins secreted by the midgut cells was collected by centrifugation (2,000× g for 20 min at 4 °C). Two independent biological samples were used with each tested in triplicate. All resulting collected secretomes (six) were pooled to prepare each secretome category (buffer or Cry1Ac), and were used either for MS/MS identification or for bioactivity assays as described below. Proteomes were stored at −80 °C until used.

Secretome samples for 1DGel LC/MS/MS analysis were concentrated to 50–100 µl and the media exchanged to 20 mM TRIS/HCl pH 8.0 buffer using centrifugal filter devices (3-kDa MWCO; Millipore, MA), following manufacturer’s instructions. Proteins in concentrated samples were quantified using the Qubit fluorometer (Invitrogen, Carlsbad, CA, USA) and then diluted to 2 mg/ml in 20 mM Tris/HCl pH 8.0 buffer. Samples were shipped to NextGen Sciences (Ann Arbor, MI, USA) for proteomic analysis.

### Stem cell bioactivity assays

Primary *H. virescens* midgut stem cell cultures were stained with the viability stain calcein AM (Invitrogen) following manufacturer’s instructions and then counted and gated using flow cytometry. Purified stem cells were seeded into individual wells of a 12-well plate at a concentration of 4 × 10^4^ cells/ml and a final volume of 500 µl. Treatments of Cry1Ac or buffer-induced secretomes were applied to the wells (final well volume was 1 ml). The time zero control was measured by adding working Grace’s media. After 30, 60 and 180 min of incubation time, proliferation and differentiation of cells were measured by staining for viability with calcein AM (Invitrogen, Carlsbad, CA, USA) and analyzed for side scatter and green (calcein AM) fluorescence by counting 20,000 non-gated events in an LSRII flow cytometer (BD Bioscience, San Jose, CA, USA), as previously described (Castagnola, Eda & Jurat-Fuentes, 2011). At least two wells for the same treatment were measured per experiment, and experiments were replicated thrice.

### Proteomic analysis of primary midgut cell culture secretome

Proteins in secretome samples (10 µg) were separated by 1D SDS-10% PAGE using the NuPAGE Bis-Tris mini gel system (Invitrogen, Carlsbad, CA, USA) following manufacturer’s instructions, and then each of the sample lanes was sliced in five cross-sections that were subjected to in-gel digestion in a ProGest workstation (Genomic Solutions, Ann Arbor, MI, USA). Briefly, samples were reduced with 10 mM DTT at 60 °C, and then allowed to cool to room temperature before being alkylated with 100 mM iodoacetamide. Tryptic digestion was done at 37 °C for 4 h, and reactions were stopped by addition of formic acid (0.1% final concentration). Analysis of peptides generated by the tryptic digestion through liquid chromatography coupled to tandem mass spectrometry (LC/MS/MS) was performed at NextGen Sciences (Ann Arbor, MI, USA) using a ThermoFisher LTQ Orbitrap XL mass spectrometer. Tandem mass spectra were analyzed at MS Bioworks (Ann Arbor, MI, USA) using Mascot (Matrix Science, London, UK) and queried against a custom *H. virescens* transcriptome database (Perera et al., 2015) translated in the 6 possible frames. Search parameters included a fragment ion mass tolerance of 0.50 Da and a parent ion tolerance of 10.0 PPM. Iodoacetamide derivative of cysteine was specified as a fixed modification, while S-carbamoylmethylcysteine cyclization (N-terminus), deamidation of asparagine and glutamine, and oxidation of methionine and acetylation of the N-terminus were specified as variable modifications.

Scaffold (version Scaffold_4.8.2; Proteome Software Inc., Portland, OR, USA) was used to validate MS/MS based peptide and protein identifications. Peptide identifications were accepted if they could be established at greater than 95% probability as specified by the Peptide Prophet algorithm (Keller et al., 2002). Protein identifications were accepted if they could be established at greater than 99.0% probability and contained at least 2 identified peptides. Protein probabilities were assigned by the Protein Prophet algorithm. Proteins that contained similar peptides and could not be differentiated based on MS/MS analysis alone were grouped to satisfy the principles of parsimony. Weighed spectral counts calculated for each protein using an internal weighting function in Scaffold that apportions shared peptides among proteins according to peptide exclusivity, were used for quantitative comparisons. Given the low number of replicates used (multiple independent secretome replicates pooled into a single sample for MS analysis for each category), we used the Fisher’s exact test, as described elsewhere (Zhang et al., 2006), to compare the relative abundance between the Buffer and Cry1Ac sample categories (Zhang et al., 2006). Significance was established at *p* < 0.005 to increase the probability of accurate detection of differences in protein levels for each of the identified proteins.

### Transcriptome profiling

Early fourth instar larvae were exposed to diet supplemented with a sublethal (no mortality after 8 h exposure) dose of Cry1Ac (1 µg/cm^2^) and observed for feeding. Midguts were dissected from actively feeding larvae after 0, 2 or 8 h and then flash frozen. Total RNA was extracted from midguts using RNAzol® RT (Molecular Research Center, Cincinnati, OH, USA) and enriched for mRNA using PolyATtract® magnetic beads (Promega, Madison, WI, USA). Approximately 500 ng of mRNA was fragmented using NEBNext® Magnesium RNA fragmentation reagents (New England Biolabs, Ipswich, MA, USA), cleaned and concentrated with MinElute Reaction Cleanup kit (Qiagen, Hilden, Germany). Double-stranded cDNA was prepared from fragmented mRNA with random hexamers using SuperScript® Double-Stranded cDNA Synthesis Kit (Invitrogen, Carlsbad, CA, USA). An Illumina TruSeq library preparation kit was used to prepare indexed libraries that were size fractionated to obtain insert lengths of 300 ± 30 bp using the LabChip XT DNA 750 Assay Kit in the LabChip XT instrument (PerkinElmer, Waltham, MA, USA). Size fractionated libraries were quantified by real time quantitative PCR using KAPA Library quantification reagents and standards (KAPA Biosystems, Boston, MA). Individual libraries multiplexed to contain equimolar concentrations were submitted to the USDA-ARS Genomics and Bioinformatics Research Unit (Stoneville, MS, USA) for sequencing (50 cycles) in an Illumina Hiseq200 platform. Sequence reads were pre-processed to remove adapters and to quality trim prior to expression profiling.

Contigs of interest based on the proteomic identification (Table 1) were selected from the transcriptome assembly of *H. virescens* (Perera et al., 2015) and used as reference sequences in expression profiling with CLC Genomics Workbench (CLC Bio-Qiagen, Aarhus, Denmark). Replicate groups were created using sequence reads from each replicate experiment. Read density for each transcript was normalized for transcript read length using the reads per kilobase of exon model per million mapped reads (RPKM) method (Mortazavi et al., 2008). The genes were then subjected to Baggerley’s test (Baggerly et al., 2003) to obtain weighted fold change of expression levels between the control and experimental replicates. The fold change values were filtered with the false discovery rate (FDR) correction of *p*-values at 0.0001 (one false discovery in 10,000 comparisons), and then normalized to an internal reference gene (Hv_Contig_26369, *β*-actin).

**Table 1:** Identified proteins with significant differences in abundance between buffer and Cry1Ac secretomes (Fisher’s Exact Test; *p* < 0.005).

Contig	AA^a^	#P^b^	Protein	*E* value	Accession #	Species	Id^c^	Cov^d^	Buffer^e^	Cry1Ac^f^	*P*^g^
997	747	26	Cry1A toxin receptor A	0.0	AAF08254.1	*Heliothis virescens*	98%	93%	67	0	<0.0001
2013	688	7	Arylphorin subunit alpha-like	0.0	AEO51737.1	*Helicoverpa armigera*	85%	96%	0	10	0.0010
2924	630	2	Uncharacterized family 31 glucosidase KIAA1161-like	0.0	XP_013174005.1	*Papilio xuthus*	71%	98%	26	53	0.0017
6027	363	7	Elongation factor 1 gamma	3e−168	NP_001298516.1	*Papilio polytes*	75%	79%	9	0	0.0019

**Notes.**

a Number of amino acids in the translated contig.

b Number of exclusive unique peptides detected for the identified protein by mass spectrometry.

c Identity percentage between the translated contig and the NCBI protein match.

d Percentage of the translated contig sequence covered by the matched NCBI protein.

e Weighted spectral counts in the buffer-induced secretome.

f Weighted spectral counts in the Cry1Ac-induced secretome.

g Fisher’s test probability estimate of comparisons between Buffer-Cry1Ac secretomes. Significance was considered at *p* < 0.005.

### Purification of alpha-arylphorin

Arylphorin levels are highest at the pre-wandering phase of the 5th larval instar in noctuid larvae (Burmester, 2015). Consequently, hemolymph was collected from 60 pharate 5th instar *H. virescens* larvae by making a small incision at the base of the 1st and/or 2nd proleg and collecting droplets into 15 mL conical tubes containing 5 mg of phenylthiourea to block hemolymph phenoloxidase activity, and maintaining on ice. After collection, hemolymph was frozen at −20 °C until used (no longer than 2 months). Frozen hemolymph was thawed on ice and diluted 5-fold in 20 mM Tris pH 7.9 (buffer A). For fractionation, hemolymph was filtered (0.22 µm) and loaded onto a HiTrap Q HP column (GE Healthcare, Little Chalfont, UK), previously equilibrated with buffer A and connected to an AKTA FPLC system (GE Healthcare, Little Chalfont, UK). Proteins were eluted with a 0–1 M linear gradient of NaCl in 20 mM Tris pH 7.9 (buffer B) at a flow rate of 1 mL/min, collecting 1 mL fractions. To reduce the presence of smaller proteins co-purifying with *α*-arylphorin, fractions estimated to contain *α*-arylphorin (based on presence of ∼70 kDa band on electrophoretic observations) were combined and filtered using an Amicon Ultra-15 mL centrifugal unit (Millipore, Billerica, MA, USA) with a MWCO of 50 kDa. After concentration, partially purified *α*-arylphorin (Fig. S1) was quantified with the Coomassie Plus Protein Assay (Pierce, Waltham, MA, USA) using BSA as the standard, and then aliquoted and maintained at −80 °C until used.

### Alpha-arylphorin feeding bioassays

Artificial diet was dispensed into wells of 128-well bioassay trays (both from Bio-Serv, Flemington, NJ, USA) and left to dry in a laminar flow cabinet. A single concentration of *α*-arylphorin observed to cause midgut hyperplasia in preliminary assays (0.781 µg/mL) was prepared in 20 mM Tris, pH 7.9 and distributed (75 µL) to the dry diet surface of each well and gently swirled to ensure even coating (29.2 ng/cm^2^ final dose per well). Upon drying, a single neonate larva was placed in each well and the wells were sealed with adhesive covers. Larvae were allowed to feed on diet containing *α*-arylphorin for five days (3rd instar) under standard rearing conditions and then moved to diet that was surface-contaminated with Cry1Ac toxin (0.5 µg/cm^2^) or toxin buffer, and mortality scored after seven days. Bioassays were conducted with 16 neonate larvae per treatment and replicated three times.

### Histological sections

The number of midgut cells in larvae fed for five days on diet supplemented with *α*-arylphorin or buffer (as described above) was determined by counting the number of nuclei incorporating DAPI (4′,6-diamidino-2-phenylindole) in gut sections. Larvae (4–6 per treatment) were fixed in ice-cold Carnoy’s solution (60% ethanol, 30% chloroform, 10% glacial acetic acid) and stored at 4 °C overnight in biopsy cassettes, and then transferred to freshly prepared 70% ethanol. Larvae were processed using a Tissue-Tek VIP processor (Sakura, Torrance, CA) and embedded in paraplast medium (Sigma–Aldrich, St. Louis, MO, USA). Block sections were obtained by cutting 5 µm slices using a Micron HM355s microtome (Thermo Scientific). For DAPI staining, tissues were mounted on Superfrost Plus Slides (Fisher Scientific, Waltman, MA, USA) following recommended guidelines (Slaoui & Fiette, 2011). Slides prepared for DAPI staining were deparaffinized through two 10 min washes in xylene followed by a rehydration series of ethanol washes (absolute, 95%, 70%) for 5 min each, and finally washed twice for 5 min in distilled water. Tissues were permeabilized by treatment with 10 mM citrate buffer pH 6.0 at 95−99 °C for 20 min and rinsed in two 1-min washes of phosphate buffered saline (PBS, 2 mM KCl,135 mM NaCl, 1.7 mM KH_2_PO_4_,10 mM Na_2_HPO_4_, pH 7.4). Blocking was performed in PBS containing 0.1% Tween-20 and 3% BSA for 1 h. After blocking, tissues were mounted in a water-based medium containing DAPI for nuclear detection. The total number of cells in each treatment was estimated by counting the number of DAPI-stained nuclei per 100 µm^2^ in 3 sections from independent larvae for each treatment. Cell size in midguts submitted to experimental or control treatments was not determined.

## Results

### Secretome bioactivity in primary midgut stem cell cultures

**Figure 1:**
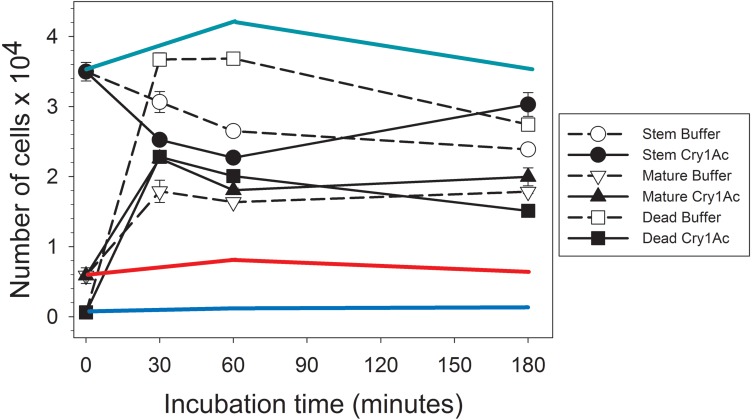
Graph showing bioactivity of secretomes on midgut stem cells. Changes in the number of *Heliothis virescens* mature, stem and dead midgut cells in primary cultures exposed to Cry1Ac or buffer-induced secretomes. Primary midgut stem cell cultures were prepared and treated with Cry1Ac or toxin buffer as a control. After the indicated, time intervals the number of cells in each cell type category (mature, stem and dead) was determined using a fluorescence-based method (Castagnola, Eda & Jurat-Fuentes, 2011). Shown are the mean number of cell types with the corresponding standard error bars calculated from four independent measurements (*n* = 4). The symbol used for each cell type and treatment is indicated in the figure. The green, red and blue lines represent estimates of the effect of Cry1Ac toxin alone on the number of stem, mature and dead cells, respectively, based on the percentage of change in cell numbers observed in independent experiments. These percentages were used to calculate the expected change in the number of cells with time based on the same initial number of stem, mature and dead cells as in the experiments with secretomes.

After collecting the proteins secreted by primary mature midgut cell cultures (secretome) in response to Cry1Ac or control buffer treatment, we determined their regenerative properties on primary *H. virescens* midgut stem cell cultures using a flow cytometry method based on differential calcein staining (Castagnola, Eda & Jurat-Fuentes, 2011). Flow cytometry gates defined by the side scattered light and calcein fluorescence allowed monitoring of changes in the number of stem, mature, and dead cell subpopulations within each sample (Fig. 1). Freshly prepared *H. virescens* midgut stem cell cultures (time zero in Fig. 1) contained predominantly stem cells (approx. 3.5 × 10^4^ cells or 85%), and a smaller proportion of mature (0.6 × 10^4^ cells or 14%) and dead (0.6 × 10^3^ or 1%) cells. Treatment with buffer-induced secretome resulted in a non-significant trend (Student’s *T*-test, *p* = 0.074) of rapid (30 min.) reduction in the number of stem cells concomitant with a significant increase in the number of dead (Student’s *T*-test, *p* < 0.05) and mature cells (Student’s *T*-test, *p* < 0.05) (Fig. 1, open symbols). Technical limitations prevented us from determining whether the cell types accounting for the increased dead cell numbers in this treatment were stem, mature or both, but this observation may reflect the low viability in culture of midgut mature cells in the original preparation and additional mature cells originating from stem cell differentiation. After three hours, only a small reduction in the number of dead cells (probably due to cytolysis) was observed in these cultures.

Similarly to treatment with buffer-induced secretome, a significant (Student’s *T*-test, *p* < 0.05) initial tendency to increase the number of dead and mature cells and decrease stem cell numbers was detected in cultures treated with Cry1Ac-induced secretome (Fig. 1, black symbols). However, an important observation was that the total number of dead cells after 30 min or 1 h was approximately half in cultures treated with Cry1Ac compared to buffer secretome treatment. After 3 h, the number of stem cells in the Cry1Ac secretome-treated samples had recovered to similar levels observed for the initial conditions, while the number of dead cells had significantly decreased compared to 30 min (Student’s *t* test, *p* < 0.05) and the number of mature cells remained constant. The recovery in the number of stem cells between 30 min and 3 h (Student’s *T*-test, *p* < 0.05) was suggestive of a regenerative response to Cry1Ac secretome compared to control treatment. This response was not observed when primary midgut stem cell cultures were treated with the same Cry1Ac toxin concentration used to obtain the Cry1Ac-induced secretome (see line estimates in Fig. 1), indicating the presence of mitogens in the Cry1Ac-induced secretome.

### Identification of secretome proteins and alterations in response to Cry1Ac intoxication

Proteins in buffer and Cry1Ac-induced secretomes from *H. virescens* primary mature midgut cell cultures were identified using LC/MS/MS and a custom *H. virescens* transcriptome database (Perera et al., 2015). A total of 358 proteins were identified in the secretomes (protein False Discovery Ratio [FDR] 0.0%), with 326 and 313 proteins detected in the buffer and Cry1Ac-induced secretomes, respectively. Out of the 358 identified proteins, 281 proteins were common to both secretomes, while 45 proteins were unique to the buffer, and 32 to the Cry1Ac induced secretomes, respectively. Normalized spectral counts for all proteins detected in the Cry1Ac secretome and their corresponding values in buffer secretome are listed in Table S1. The list of identified proteins included proteins involved in physiological functions expected for the gut tissue, such as digestive enzymes, storage and transport proteins, immune and stress-related proteins, and also putative Cry toxin receptors. Intracellular proteins (ribosomal, mitochondrial and cytosolic enzymes, nucleotide-related proteins) were also commonly detected, possibly due to leakage of cell contents after cell death.

Statistical analyses of weighed spectral counts identified 4 proteins with significant different abundance (Fisher’s Exact test with Benjamini–Hochberg correction; *p* < 0.005) when comparing buffer to the Cry1Ac-induced secretome (Table 1). Differentially present proteins included digestive enzymes (aminopeptidase and glucosidase), a protein involved in processes of cell growth and proliferation (elongation factor 1 gamma), and a storage protein (arylphorin subunit alpha). Both the Cry1A receptor aminopeptidase and the elongation factor 1 gamma were unique to the buffer secretome, while only *α*-arylphorin was unique to the Cry1Ac secretome. An uncharacterized glucosidase was more abundant in the Cry1Ac secretome (Table 1).

**Figure 2:**
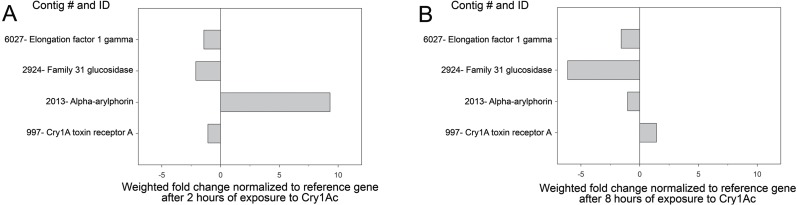
Expression of identified contigs from proteomic analysis after Cry1Ac intoxication. Analysis of transcript levels detected for contigs detected as differentially present proteins in the buffer-Cry1Ac secretome comparison. Shown are fold change in transcript levels for each contig in midguts from *H. virescens* larvae after 2 (A) or 8 (B) hours of exposure to Cry1Ac compared to initial levels. The vertical line at “0” indicates no change in transcript levels.

Transcriptome profiling in larvae exposed to Cry1Ac for 2 and 8 h was used to confirm the levels of expression of the contigs matching to the proteins identified as differentially present in the buffer- and Cry1Ac-induced secretomes. We chose these time points as an early (2 h) and late (8 h) times in the Cry1Ac intoxication process based on previous reports (Forcada et al., 1996; Spies & Spence, 1985). When fold difference in expression levels after 2 h of exposure to Cry1Ac was estimated using the RKPM method (Mortazavi et al., 2008), we observed reduced expression of contigs 997 (aminopeptidase), 6027 (elongation factor), and 2924 (glucosidase), while expression of *α*-arylphorin was increased more than 9-fold (Fig. 2), in agreement with the proteomic findings. In contrast, after 8 h of exposure to Cry1Ac, only contig 997 had slightly increased expression, while all other three contigs had reduced expression (Fig. 2). When expression of arylphorin contigs detected in the *H. virescens* transcriptome was examined, we observed that a group of related arylphorin contigs was up-regulated in response to Cry1Ac, while a second group did not respond or had reduced expression levels (Fig. S2). Contig 2879 (*α*-arylphorin) detected in the secretome analysis and that was also unique to the Cry1Ac secretome (not included in Table 1 since *p* < 0.016), also displayed increased expression after exposure of larvae to Cry1Ac (Fig. S2).

Interestingly, when comparing levels of expression of arylphorin contigs in the Hv transcriptome (Perera et al., 2015) after exposure to Cry1Ac (Fig. S2), we found differences in expression among arylphorin contigs that were distantly related. For instance, in contrast to contigs matching to arylphorin subunit alpha being up-regulated, contig 19307 matching to arylphorin subunit beta of *H. armigera* (accession XP_021195808.1; *E*-value 2.00E−90) was down-regulated after exposure to Cry1Ac. Contig 35193 matching with 74% sequence identity to arylphorin from *H. armigera* (accession AEO51737.1; *E*-value 5.00E−40) did not display drastic changes in expression after larvae were exposed to Cry1Ac.

**Figure 3:**
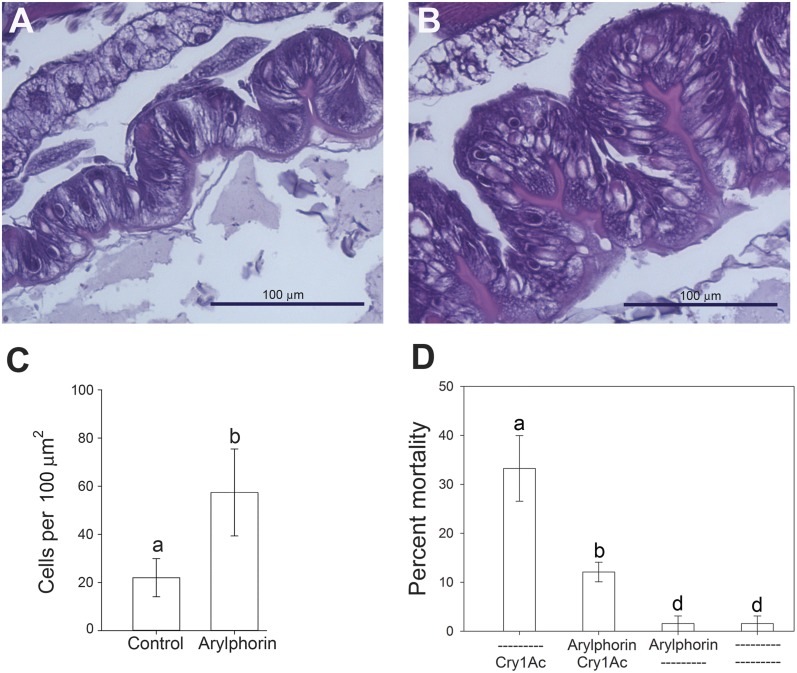
Effect of feeding on arylphorin on midgut hyperplasia and susceptibility to Cry1Ac. Testing of the mitogenic effect of *α*-arylphorin in *H. virescens* larvae and its effect on susceptibility to Cry1Ac. Histological examination of midgut epithelial tissues from *H. virescens* larvae after feeding for 5 days on diet supplemented with control buffer (A) or 29.2 ng/cm^2^ of purified *α*-arylphorin (B). After midgut dissection, tissues were embedded and stained with hematoxylin and eosin. (C) Total number of cells per 100 µm^2^ of midgut epithelial tissue in control or after treatment with 29.2 ng/cm^2^ of *α*-arylphorin for 5 days, as indicated. Small bars denote standard error of the mean for each treatment obtained from sections counted from three independent midgut tissues; different letters for each column denote statistically significant differences (*p* < 0.05, Student’s *t*-test) among treatments. (D) Percentage mortality of *H. virescens* larvae exposed to control (–) or diet containing 29.2 ng/cm^2^ of purified *α*-arylphorin on its surface (Arylphorin) for five days, and then to diet containing 0.5 µg/cm^2^ Cry1Ac toxin (Cry1Ac) for seven days. Bars denote standard error of the mean for each treatment of 16 larvae and calculated from three bioassay replicates; statistically significant differences between treatment and control groups are denoted by different letters for each column (*p* < 0.05, One-way ANOVA).

### Effect of *α*-arylphorin on *H. virescens* susceptibility to Cry1Ac toxin

Given that *α*-arylphorin was the only protein unique to the Cry1Ac-induced secretome among the differentially present proteins between the buffer and Cry1Ac-induced secretomes, we concentrated our efforts on the putative role of this protein in the midgut response to Cry1Ac intoxication. To test the effect of *α*-arylphorin during the response to Cry1Ac intoxication, we purified arylphorin from *H. virescens* hemolymph and fed it to neonates for five days before exposing them to Cry1Ac toxin. Mass spectrometry and electrophoretic analyses of the purified *α*-arylphorin sample supported that it was >87% pure (Fig. S1). Larvae feeding on this purified *α*-arylphorin sample for 5 days developed midgut hyperplasia when compared to controls (Fig. 3A). This hyperplasia resulted from a significant increase in the number of cells per unit of midgut surface (Fig. 3B). Exposure of larvae fed buffer without *α*-arylphorin to Cry1Ac toxin (0.5 µg/cm^2^) for seven days resulted in 34 ± 5% mortality. In contrast, a significant reduction (Student’s *t*-test; *p* < 0.05) in mortality to 15 ± 3% was detected in larvae that had been pre-exposed to *α*-arylphorin (29.2 ng/cm^2^) before exposure to Cry1Ac (Fig. 3C). No significant difference was detected between mortality in control larvae (3% mortality) and larvae exposed to *α*-arylphorin for five days (Fig. 3D) (Mann–Whitney Rank Sum Test used due to data failing normality test; *p* < 0.05).

## Discussion

Insect gut cells are responsible for the absorption of nutrients and secretion of proteins into the gut lumen to help regulate digestion. Previous studies on lepidopteran larval midgut proteomes have focused on proteins present in the peritrophic matrix (Campbell et al., 2008) and midgut lumen (Pauchet et al., 2008), or the identification of putative binding sites for Cry insecticidal proteins (Krishnamoorthy et al., 2007; McNall & Adang, 2003). In this study we aimed to help resolve the current lack of proteomic information on the response to noxious stimuli, more specifically the Cry1Ac toxin, in the lepidopteran midgut cells. We report on the identification of proteins secreted by primary larval midgut cell cultures of *H. virescens* and their differential secretion of proteins after exposure to Cry1Ac toxin, the most active Cry toxin reported against that insect (Van Frankenhuyzen, 2009). Even though a limited number of biological replicates was used, confirmation of data from six pooled secretomes with transcriptome profiling analysis support increased expression of *α*-arylphorin, but not *β*-arylphorin, after exposure to Cry1Ac.

Primary *H. virescens* midgut cell cultures were described to undergo a regenerative process after exposure to a Cry toxin, which involved an increase in the number of differentiating cells compared to controls (Loeb et al., 2001a). This process was observed in our experiments after treatment with Cry1Ac-induced secretome as an increase in the number of mature cells concomitant with an initial decrease in the number of stem cells, consistent with stem cell differentiation, followed by an increase in the number of stem cells to initial levels (evidence of stem cell proliferation). In contrast, the proliferative stem cell phase was not observed when treating the cell cultures with the secretome induced by buffer treatment. Based on these observations, we hypothesized the presence of growth factors in the Cry1Ac-induced secretome implicated in the regenerative process.

Analysis of the control and Cry1Ac-induced secretomes identified proteins expected to be produced and secreted by enterocytes (Table S1), including digestive enzymes (proteases, glucosidases and lipases), storage and transport proteins (transferrin, apolipophorin, arylphorin, fatty acid-binding protein...), proteins involved in defense reactions (phenoloxidase, esterases, glutathione S-transferases...), and enzymes involved in diverse gut physiological processes (phosphatases, dehydrogenases, deaminases...). The relevant abundance of putative intracellular proteins detected in both secretomes probably represents the release of cellular contents into the media after enterocyte death. Proteins unique to the buffer secretome included Cry1A toxin receptor aminopeptidase and elongation factor 1 gamma. Shedding of GPI-anchored proteins such as aminopeptidases from the midgut cell surface has been previously reported in midgut cells of *Lymantria dispar* after exposure to Cry1Ac, probably as a way to reduce available levels of *Bt* receptors on the midgut cell surface (Valaitis, 2008). This observation is in contrast to the increased Cry1A toxin receptor aminopeptidase levels observed in the buffer secretome. While it needs to be considered that the epithelial polarization missing in primary cell cultures may affect which GPI-anchored proteins are shed, we currently do not have an explanation for the unique shedding of a putative Cry1Ac receptor aminopeptidase after treatment with control buffer but not during exposure to Cry1Ac. One possibility could be that the shedding of selected GPI-anchored proteins may be a natural process independent of exposure to Cry proteins.

It is also challenging to interpret the detected changes in abundance for other proteins, such as family 31 glucosidase and elongation factor 1 gamma. Increased glucosidase levels during exposure to Cry1Ac compared to buffer may reflect an increased catabolism in cells exposed to Cry proteins. This hypothesis is supported by the increased expression of glycosyl hydrolases and other enzymes involved in catabolism reported for *Tenebrio molitor* larvae exposed to Cry3Aa (Oppert et al., 2012). In contrast, reduced expression of catabolic enzymes has been reported after exposure of lepidopteran larvae to Cry toxins (Van Munster et al., 2007), including levels of a glycosyl hydrolase family 31 protein in *Spodoptera frugiperda* after exposure to Cry1Ca toxin (Rodriguez-Cabrera et al., 2008). The discrepancy between observations described here and previous reports in Lepidoptera may be related to the differences between *in vivo* and *in vitro* systems used in analyzing responses to Cry toxins. In line with the proposed increased catabolism, the reduced levels of elongation factor 1 gamma protein in Cry1Ac secretome may be indicative of reduced protein biosynthesis, although a role in cytoskeleton reorganization has also been proposed for this protein (Shiina et al., 1994). Levels of elongation factor 1 gamma are increased in actively proliferating cells, such as those in culture (Sanders, Maassen & Moller, 1992) or in gastric tumors (Mimori et al., 1995). Consequently, it is possible that the reduced levels detected for this protein in the Cry1Ac compared to buffer secretome represent a reduction in anabolism concomitant with the proposed increased catabolism in cells exposed to Cry proteins.

Of the four proteins identified to be differentially present in buffer or Cry1Ac induced secretomes, *α*-arylphorin was the only protein unique to the Cry1Ac secretome, suggestive of a relevant role in response to intoxication. Transcriptome profiling supported a highly significant increase in the expression of selected *α*-arylphorin contigs, including contig 2013 detected in the secretome analysis, after 2 h of exposure to Cry1Ac. In contrast, *β*-arylphorin and other arylphorin contigs with <70% sequence identity to *α*-arylphorin displayed reduced or not changed expression. This differential expression is probably evidence for diverse functional roles (transport vs. immunity) of genes in this family. During the 2 h of exposure to Cry1Ac it is expected that the intoxication process has started to cause relevant damage to the midgut epithelium, so that a healing response is activated. Moreover, and while speculative at this point, lower expression of the *α*-arylphorin gene at 8 h of exposure probably reflects tight regulation of arylphorin as participant in the midgut healing response. Further work is necessary to test this hypothesis and determine the specific role of arylphorin in the insect midgut defensive response.

While traditionally considered a storage protein produced by the fat body, arylphorin has been shown to stimulate midgut stem cell proliferation (Blackburn et al., 2004; Hakim et al., 2007; Smagghe et al., 2005), and its production in the lepidopteran midgut epithelium is also established (Palli & Locke, 1987; Tang, Wang & Zhang, 2010). Moreover, there is growing evidence supporting a role for arylphorin in insect immunity. For instance, increased *α*-arylphorin gene expression was previously detected in response to infection with bacteria (Freitak et al., 2007) and parasites (Kunkel et al., 1990) in larvae of *Trichoplusia ni*. These increased *α*-arylphorin levels could result in midgut hyperplasia, as previously described (Smagghe et al., 2003) and as observed in the present study. Increased levels of hexamerins, such as arylphorin were proposed to sequester Cry1Ac toxin to the gut lumen in *H. armigera* (Ma et al., 2005). In contrast, reduced levels of arylphorin transcripts were detected upon exposure of *S. exigua* to Vip3Aa or *L. dispar* to *Bt* var. *kurstaki* (Bel et al., 2013; Sparks et al., 2013). These observed discrepancies in the regulation of arylphorin may represent differences in the mode of action of Cry versus Vip3Aa toxins and the effect of *Bt* spores versus purified Cry proteins on midgut cells. Alternatively, it is possible that the mitogenic effect of *α*-arylphorin depends on its relative concentration, as previously reported (Hakim et al., 2007).

Our data from feeding bioassays support that midgut hyperplasia induced by *α*-arylphorin is relevant to increase survival during exposure to Cry1Ac. Increased *α*-arylphorin expression was also detected in *S. exigua* resistant to a *B. thuringiensis* pesticide, yet this increase was not concomitant with enhanced midgut regeneration (Hernández-Martínez et al., 2010). Given that enhanced midgut regeneration was previously hypothesized as a resistance mechanism to Cry1Ac in *H. virescens* (Forcada et al., 1999; Martínez-Ramírez, Gould & Ferré, 1999), the potential involvement of arylphorin in this response needs to be further evaluated.

The identification of genes involved in the insect midgut defensive response to pathogens allows the development of strategies aimed at hindering this response for insecticidal use. The present study provides a first list of proteins that are differentially released by midgut cells *in vitro* in response to Cry1Ac intoxication. *In vivo* evidence is also provided for the relevance of one of the identified proteins, *α*-arylphorin, in defense against Cry1Ac intoxication. Given the observed mitogenic effect of *α*-arylphorin on midgut stem cells, the role of this protein in defense against infection with alternative pathogens or xenobiotics affecting the midgut epithelium is predicted.

##  Supplemental Information

10.7717/peerj.3886/supp-1 Data S1 Raw data for all figures in manuscript

10.7717/peerj.3886/supp-2 Data S2 Supplementary figures 1 and 2

10.7717/peerj.3886/supp-3 Table S1 Proteins identified in primary midgut cell secretomes

## Additional Information and Declarations

### Data Availability

The following information was supplied regarding data availability: The raw data has been supplied as a Supplementary File.
