# Supplementary material for: Alpha-arylphorin is a mitogen in the Heliothis virescens midgut cell secretome upon Cry1Ac intoxication
Source: PeerJ. 2017 Oct 3;5:e3886. doi: 10.7717/peerj.3886 (PMC5629956; doi:10.7717/peerj.3886)
Supplement: Data S2 [file peerj-05-3886-s002.docx]

Supplementary Data

Arylphorin is a mitogen in the *Heliothis virescens* midgut cell secretome upon Cry1Ac intoxication

Castagnola^1§^, A., Jackson^2§^, J., Perera^3^, O. P., Oppert^1§^, C., Eda^4^, S., and J. L. Jurat-Fuentes^1*^

^1^Department of Entomology and Plant Pathology, University of Tennessee, Knoxville, TN 37996, USA

^2^Genome Science and Technology Program, University of Tennessee, Knoxville, TN 37996, USA

^3^United States Department of Agriculture- Agricultural Research Service, Southern Insect Management Research Unit, Stoneville, MS, 38776, USA

^4^Department of Forestry, Wildlife, and Fisheries, University of Tennessee, Knoxville, TN 37996, USA

^§^Current address: A.C.: ManTech International Corporation, Herndon, VA 20171, USA; J.J.: Oklahoma State University, Department of Microbiology and Molecular Genetics, Stillwater, OK 74078, USA; C.O.: Bayer CropScience, Morrisville, NC 27560, USA


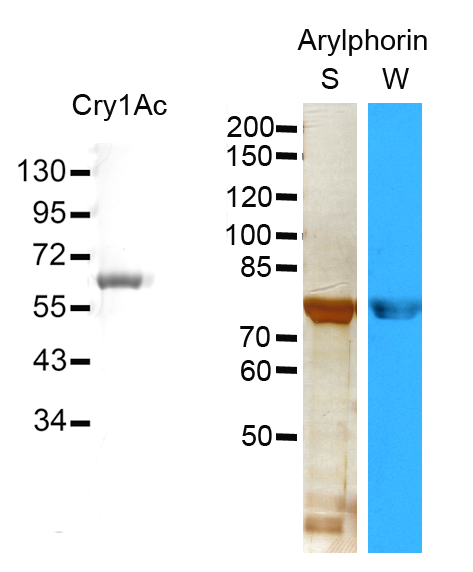


**Supplementary Figure 1**.- Purified Cry1Ac and arylphorin used in this work. Both proteins were resolved by SDS-10%PAGE and then detected by staining with Coomassie (Cry1Ac) or silver staining (Arylphorin S). Purified arylphorin was transferred to PVDF filters as described elsewhere (Perera et al., 2009), and blocked filters probed with a 1:10,000 dilution of antisera against the p76 arylphorin fragment (kindly provided by Dr. Kent Shelby, USDA-ARS Biological Control of Insects Research Laboratory, Columbia, MO). After probing with a 1:45,000 dilution of anti-rabbit HRP-conjugated antisera, the cross-reactive band (Arylphorin W) was detected using enhanced chemiluminescence (SuperSignal West Pico substrate, Pierce).

**
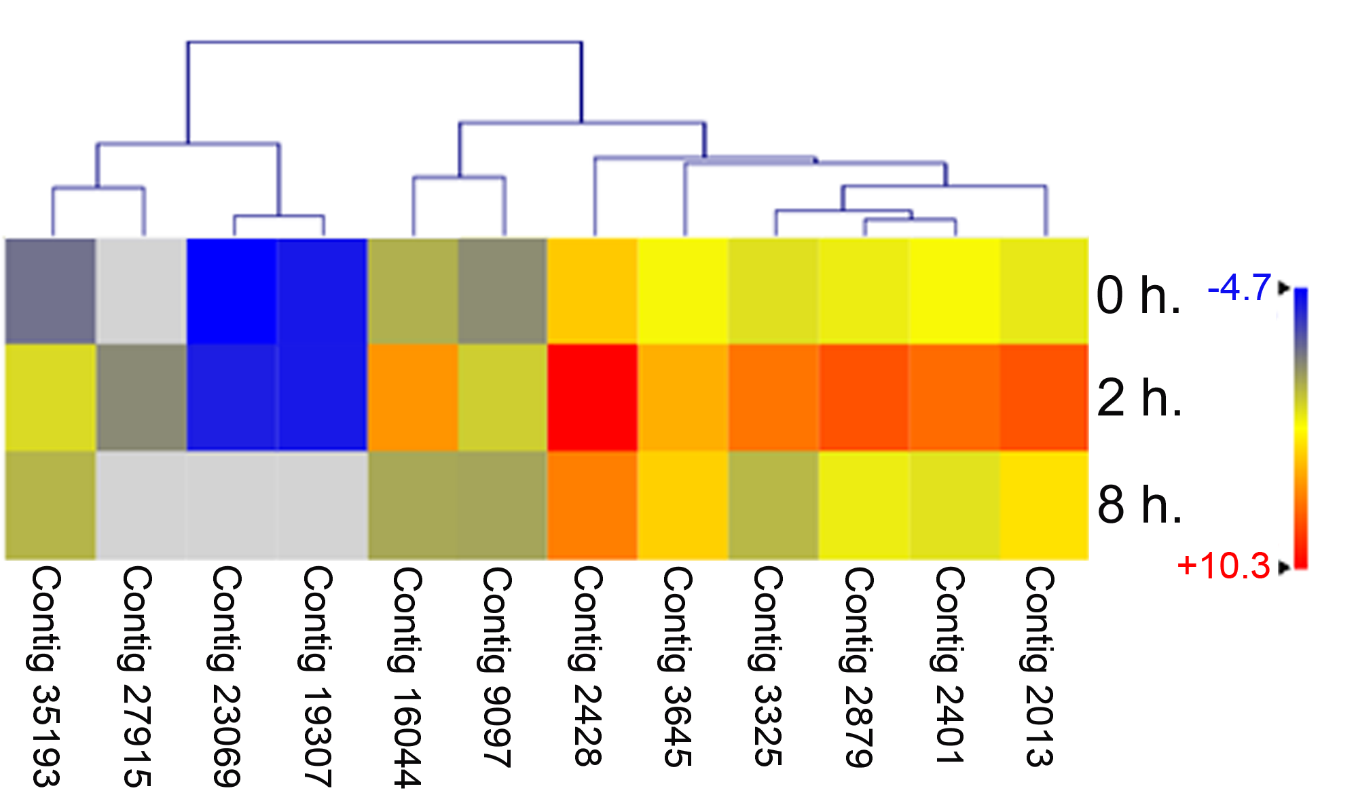
**

**Supplementary Figure 2.** Heat map for the expression of arylphorin contigs in the *H. virescens* transcriptome (Perera et al., 2015) compared to expression of an internal reference gene (Contig 26369, β-actin). Contigs were classified in a phylogenetic tree according to their sequence identity and their relative levels of expression displayed as fold expression level according to the color code shown at the right of the figure. Expression was monitored at three time points (0-2-8 hours) of exposure to Cry1Ac toxin. Details of the RNAseq sequencing and transcriptome analysis are presented in the Materials and Methods section.

**References cited**

Perera, O.P., Shelby, K.S., Popham, H.J., Gould, F., Adang, M.J., Jurat-Fuentes, J.L., 2015. Generation of a transcriptome in a model lepidopteran pest, *Heliothis virescens*, using multiple sequencing strategies for profiling midgut gene expression. PLoS One 10, e0128563.

Perera, O.P., Willis, J.D., Adang, M.J., Jurat-Fuentes, J.L., 2009. Cloning and characterization of the Cry1Ac-binding alkaline phosphatase (HvALP) from *Heliothis virescens*. Insect Biochemistry and Molecular Biology 39, 294-302.
